# Nonalcoholic Fatty Liver Disease: A Challenge from Mechanisms to Therapy

**DOI:** 10.3390/jcm9010015

**Published:** 2019-12-19

**Authors:** Giovanni Tarantino, Vincenzo Citro, Domenico Capone

**Affiliations:** 1 Department of Clinical Medicine and Surgery, “Federico II” University Medical School of Naples, 80131 Naples, Italy; 2 Department of General Medicine, “Umberto I” Hospital, 84014 Nocera Inferiore (Sa), Italy; v.citro@libero.it; 3 Care Department of Public Health and Drug-Use, Section of Medical Pharmacology and Toxicology, “Federico II” University, 80131 Naples, Italy; docapone@unina.it

**Keywords:** NAFLD, NASH, obesity, ROS, IR, inflammation

## Abstract

Focusing on previously published mechanisms of non-alcoholic fatty liver disease (NAFLD), their uncertainty does not always permit a clear elucidation of the grassroot alterations that are at the basis of the wide-spread illness, and thus curing it is still a challenge. There is somehow exceptional progress, but many controversies persist in NAFLD research and clinical investigation. It is likely that hidden mechanisms will be brought to light in the near future. Hereby, the authors present, with some criticism, classical mechanisms that stand at the basis of NAFLD, and consider contextually different emerging processes. Without ascertaining these complex interactions, investigators have a long way left ahead before finding an effective therapy for NAFLD beyond diet and exercise.

## 1. Introduction

Obesity, dyslipidemia, hypertension, and type 2 diabetes mellitus (T2DM) are mostly prominent in western societies, and together represent the well-known metabolic syndrome. Focusing on obesity, there is a great body of evidence that both genetic and environmental factors are important in its genesis and evolution. Whilst the genetic factors are still poorly understood, numerous studies have shown that for abdominal obesity in particular, physical inactivity is central. In this sense, obesity is a major risk factor for the development of insulin resistance (IR), a key factor in the etiology of metabolic syndrome and T2DM [1]. In turn, IR promotes the development of non-alcoholic fatty liver disease (NAFLD), which in a subset of patients could evolve to a more severe form, i.e., non-alcoholic steatohepatitis (NASH). NASH is characterised by fatty infiltration of the liver and inflammation, leading to the apoptosis and necrosis of hepatocytes, features to which reparative responses follow, resulting in fibrosis [2]. Unfortunately, science is far from completely clarifying the genesis of these diseases (NAFLD/NASH) and suggesting appropriate therapy to drastically reduce or eliminate them. Furthermore, there is still much debate as to whether NAFLD/NASH is a primary or secondary illness, i.e., linked to obesity and T2DM. Nowadays, significant progress has been made to decrease the burden of such diseases, although there is long way to go. Looking at each type of treatment, i.e., causal treatment; prophylactic treatment; symptomatic treatment [3], we cannot help stressing that the most appropriate is only the first one. However, causal treatment requires a complete understanding of casual factors and subsequent mechanisms. Where does the research on this topic stand? What definitive casual relationships have been found, if any?

The supposed mechanisms that are at the basis of numerous attempts to cure NAFLD, until now have ended, at best, in modest results. In recent years, we have faced many interesting studies, but few are conclusively accepted by experts. We try to provide evidence of the complexity, and for some aspects the contradictions, of the main lines of basic and clinical research addressing this topic.

## 2. The Role of Reactive Oxygen Species (ROS) Overproduction

Malnutrition splits in two directions; undernutrition and overnutrition; both of them leading to severe health complications: Famine and overweight/obesity are the result of calorie deficit or excess, and both of them are major health problems. Constantly eating excess calories leads body fat stores to expand. One of the most active and productive hypotheses about the mechanisms inducing or worsening obesity and consequently NAFLD is the over-production of reactive oxygen species (ROS). Oxidative stress accompanying obesity is reckoned to be a key factor in the development of insulin resistance (IR), [4]. In fact, obese mice treated with NADPH oxidase inhibitors (reducing ROS) have clearly demonstrated the involvement of ROS in the genesis of IR, hepatic steatosis, and T2DM [5]. Mitochondria are clearly considered to be main contributors to oxidative stress in obesity induced by chronic over-nutrition, with subsequent reduced insulin sensitivity or IR [6]. The trigger that causes the oxidative stress in obesity is the energy surplus that eventually generates reducing equivalents. These exceed the rate of ATP utilization, giving space to the over-production of O_2_, and consequently reducing the redox-buffering capacity [7]. The aforementioned pathway has been fully highlighted in mice, characterized by deletion of manganese superoxide dismutase at the adipocyte level through enhanced mitochondrial biogenesis, mediated by uncoupling protein 1 [8].

It has previously been stressed that excessive ROS production in obesity causes IR. In fact, the presence of high ROS levels, activates the c-Jun N-terminal kinases through various mechanisms (inflammatory cytokines and free fatty acids), [9]. However, some unexpected data favor the opposite, indicating that ROS is required for insulin secretion by β-cells as well as insulin sensitivity [10].

On the contrary, in another malnutrition status, such as anorexia nervosa, liver enzymes may increase as part of the re-feeding process due to liver fat storage, and can be distinguished from starvation hepatitis by the finding of hepatic steatosis on ultrasonography [11].

## 3. Polymorphisms and NAFLD

Patatin-like phospholipase domain-containing protein 3 (PNPLA3) (PNPLA3 p.I148M polymorphism), expressed in the liver and easily discovered in subcutaneous adipose tissue of obese patients, also called adiponutrin, has been involved in the pathogenesis of NAFLD [12,13,14], as well as in the alcoholic form [15,16]. However, this genetic accumulation of liver fat does not increase the burden of subclinical atherosclerosis, and consequently of cardiovascular (CV) disease, unlike metabolic syndrome-linked NAFLD [17]. This lack of CV risk is surprising in light that laparoscopic gastric banding (LAGB) of severely obese patients decreases body weight and inflammation of the liver and adipose tissue. Furthermore, in this study, authors found that weight loss induced by LAGB impacted on PNPLA3 expression in both the liver and in fat. Weight loss following LAGB brings the tissue expression of PNPLA3 back to normal, which is reduced by TNF-α [18].

In addition to this, when dealing with polymorphisms, researchers should consider the following aspect: Taking into account that the genetic basis of adaptation to different environments is central to evolutionary biology, polymorphism is quite a normal process in nature, related to biodiversity, genetic variation, and adaptation. Its purpose is to retain variety of form in a population living in a varied environment [19], according to the motto “variety is the spice of life”.

## 4. Adipose Tissue Expandability

The adipose tissue expandability hypothesis states that a failure in the capacity for adipose tissue expansion, rather than obesity, per se, is the key factor linking positive energy balance and T2DM with related diseases. All individuals are given a maximum capacity for adipose expansion, which is determined by both genetic and environmental factors. Once the limit of adipose tissue expansion is reached, adipose tissue ceases to store energy efficiently and lipids begin to accumulate in other tissues. Ectopic lipid accumulation in non-adipocyte cells causes lipotoxic insults including IR, apoptosis, and inflammation [20].

The concept of allostatic overload begins when there is sufficient or even excess energy consumption. In this case, secretion of glucocorticosteroids and activity of other mediators of allostasis, such as the autonomic nervous system, CNS neurotransmitters, and inflammatory cytokines, wax and wane with allostatic load. If the allostatic load is persistently high, diseases occur. This mechanism could be at the basis of how obesity, T2DM, and NAFLD develop, and explain the inflammatory response linked to adipose tissue expandability. In keeping with this hypothesis stands a novel study. In high fat diet (HFD) all white adipose tissue (WAT) depots of C57BL/6J mice adipocytes augmented during HFD feeding, adipocyte enlargement is a generic response. Interestingly, in week six, when the adipocytes of epididymal WAT (eWAT) rapidly reached a maximum size, the first crown-like structures; composed of macrophages surrounding dead or dying adipocytes, as a histologic hallmark of the pro-inflammatory process observed in eWAT; greatly rose in week 12 when the maximum capability of eWAT likely achieved the overgrowth and broadest volume [21].

As previously reported, in order to maintain a normal energy homeostasis, as a consequence of calories excess, a regular fat mass expansion is critical but not always detrimental. Beyond the storage of triglycerides in visceral and subcutaneous adipose tissue, there is a similar deposition of other lipophilic compounds, i.e., endocrine disrupting compound (EDCs), also called environmental contaminants, that play a main role in the etiology of obesity and its related metabolic disorders. This bioaccumulation could impact on adipose tissue function, but adipose tissue in this case functions as a reservoir for EDCs, saving other organs from damage from these compounds.

Specifically assessing the toxicity of the organophosphates diazinon and parathion on the development of neural structures, the adverse effects were partially counteracted by the consumption of a HFD, suggesting that sequestration of EDCs in fat likely executes a protective role in limiting their toxicity, and function as an adaptive mechanism [22,23,24].

Some studies in mouse models have ascertained that adipose tissue could turn out to be hypoxic during adipose expansion in the case of obesity. The decreased oxygen levels within adipose tissue induce the hypoxia inducible factor (HIF) signalling cascade, and successively an up-regulation of pro-inflammatory feedbacks, which end up in IR. Despite this, genetic models addressing different components of the HIF pathway have yielded unclear results [25].

## 5. Environmental Factors

Some environmental factors might contribute to the development of ectopic fat storage. Climate change and adaptive nutrition are central factors due to adipose tissue’s substantial capacity to impact energy expenditure and thermogenesis, and the related decreases in seasonal cold exposure.

Decreased diet-induced thermogenesis due to a highly palatable high-fat and high-sugar diet and modest physical activity provide for the preponderance of obesity and metabolic syndrome. The most important factor that should be studied with regards to the cause of obesity is not energy expenditure, but the gradual modification of energy balance and the power to regulate body energy stores. Now, considering that a rapid increase of obesity has arisen during this time of rapid environmental and cultural change, a deep reflection on the behavioural and environmental effects on regulation of energy balance is necessary to try to elucidate the pathogenesis of NAFLD [26].

We are witnessing dramatic changes in climate. In fact, global warming, basically caused by emissions of too much carbon dioxide, is a fact. This situation is going to produce more extreme weather events with torridity and alluvion. There is evidence of a relationship between thermal exposure and the prevalence of obesity, with much more time being spent in the thermal comfort zones. Long term exposure to the thermal comfort zone might decrease brown adipose tissue, characterised by the expression of the thermogenic uncoupling protein 1 [27].

Thermogenesis is an important player in both whole-body energy expenditure and metabolic homeostasis. Recently, authors discovered that Tsukushi (TSK) is a liver-enriched secreted molecule that is highly inducible following increased energy expenditure. Hepatic TSK expression and plasma TSK levels were elevated in obese patients. In mice, TSK deficiency augmented sympathetic innervation and norepinephrine release in adipose tissue, ending up in an amplification of adrenergic signalling and thermogenesis, a modest reduction in brown fat whitening, and the related maintenance of diet-induced obesity [28].

## 6. Spleen

There is a controversial role of the spleen (Table 1): Is it an inducer or protective organ of NAFLD? The link between IL-6 and spleen enlargement is hypothesised by the results of a study performed in patients suffering from the more severe form of NAFLD, i.e., NASH, diagnosed by histology. Evaluating spleen volume by ultrasound, NASH patients showed higher values of spleen longitudinal diameter (SLD) as well as significantly higher IL-6 and vascular endothelial growth factor concentrations than in the benign form of steatosis, i.e., fatty liver. Splenic artery resistive index (index of organ damage) overlapped between patients with NASH and those with fatty livers, but showed a difference to controls, pointing out that NASH patients did not present increased liver injury (followed by fibrosis and eventual portal hypertension) compared to fatty liver patients [29]. With regards to IL-6, most interestingly, recent results showed that different sex hormone-related interventions affected the severity of NASH, likely by impacting on the level of sex hormones and modulating the Toll-Like Receptor-MyD88-IL-6 signalling pathway, clearly reinforcing the hypothesis that IL-6 plays a key role in worsening the simpler fatty liver disease [30]. Finally, in a large, multi-ethnic population with NAFLD, IL-6 was independently associated with the prevalence and severity of subclinical atherosclerosis [31]. In a milestone study in histologically diagnosed NASH patients with mild fibrosis (stages 1–2), spleen enlargement was shown to be a clear finding in NASH, especially in the early-stages [32]. Similarly, an increased spleen volume has been found in NASH patients as result of a study designed retrospectively to evaluate the utility of sonographic parameters compared to histology, for differentiating simple steatosis from NASH. Authors concluded that spleen size, assessed as SLD, was an independent predictor of NASH [33]. An interesting study performed in Tsumura Suzuki Obese Diabetes mice; a well-established model of obese NASH, including microvesicular fatty degeneration, hepatocellular ballooning degeneration, Mallory bodies, and inflammatory cell infiltration; showed that accumulation of splenic iron may synergistically occur during the progression of NASH. In addition, the authors showed that splenic iron level was significantly associated with the severity of NASH, confirming the main role of the spleen [34]. A way to explain the role of iron overload in the pathogenesis and progression of early-stage NASH is that iron homeostasis is controlled by hepcidin (Hpc). Authors studying the modulatory role of inflammatory agents (IL-6 and lipopolisaccaride, (LPS)) on Hpc in HepG2 cells preloaded with Fe, found that Hpc expression is regulated by both iron storage and inflammation, hypothesizing an inverse control in this hepatic cellular model to preclude excessive increases in Hpc [35]. These data further lend credence to the central role of the spleen, but also point out a special link between the spleen and IL-6. This is one of the various and sometimes unexplored associations. In fact, the pathogenesis of NASH, in particular the mechanisms of its onset, is the consequence of a complex interplay between host and environmental factors, and is under intense investigation. Genetic factors, biochemical factors, and abnormalities in immunological aspects and hormonal cascades play a certain role. In this complex contest, the pro-inflammatory state that shifts the body toward a more lipogenic profile favors progression toward IR and fibrosis of NAFLD. The low-grade chronic inflammation is triggered by substances derived by adipose tissue (mainly visceral) and the spleen. To try and clarify the mechanisms that are at the basis of this pro-inflammatory profile, authors examined events leading up to crosstalk between adipocytes and immune cells. Studying isolated spleen-derived immune cells stimulated with LPS, together with cultured adipocytes, the researchers selected the effects of paracrine factors and cell–cell contact on IL-6 secretion levels and secretion profiles. When splenocytes and adipocytes were co-cultured without direct contact, allowing only paracrine interaction, secretion of IL-6 was increased by three times compared to what was secreted by single cultures, showing the mediating role of the spleen [36].

In contrast, authors found a protective role of the spleen in the determination of steatohepatitis in HFD-induced obese rats. Male Sprague-Dawley rats were fed HFD and split into two groups; a splenectomy (SPX) group and a sham-operation (Sham) group. Hepatic tissue and WAT were removed one and six months after surgery, and the effects of SPX on WAT and HFD-induced fatty liver were studied. SPX rats showed exacerbated dyslipidemia and inflammation in WAT one month after surgery. Hepatic steatosis and inflammation were accelerated by SPX. At one month after surgery, the tissue triglyceride content clearly augmented in SPX rats versus Sham controls. Hepatic histology demonstrated a worsening of steatosis in obese rats. At six months after SPX, increased inflammation and fibrosis were found in liver tissue sections. WAT and liver tissue levels of inflammatory markers such as TNF-α, and the expression of Kupffer cells, were amplified at the same time in SPX rats, compared to Sham controls. These interesting results indicate that the preservation of the spleen contributes to the prevention of the progression of hepatic steatosis to steatohepatitis in obese rats [37]. Thus, does the spleen play a protective role, or does not it?

Body weight decrease resulting from exercise and diet-control in potential living liver donors is effective in reducing fatty liver. Decreased percentages of the fatty content and volume of the liver was observed in these donors. The volumes of the right and left lobes of the liver decreased significantly after body weight reduction. But what about the spleen? The volume of spleen did not change [38], casting doubts on the role of lifestyle on immune processes. In contrast, up to date data reinforce the concept that the immune system is a sensor of the metabolic state, showing a link between hepatic growth factor levels and basal metabolic rate, which is mediated by IL-16 (cytokine promoting important inflammatory pathways), and pointing out the influence of the spleen, as a main immune organ [39]. To test whether hepatic and splenic metabolic activities were linked, the glucose uptake rate per unit tracer distribution volume, i.e., metabolic rate of glucose (MRglu), evaluated by dynamic PET, was performed. MRglu was found to be higher in patients with hepatic steatosis than without, and correlated inversely to hepatic computerised tomography (CT) density. In men, SLD correlated significantly to the hepatic CT density of hepatic MRglu and splenic MRglu. Of interest, Splenic Ki/V(0), which represents tissue phosphorylated ^18^F-fluorodeoxyglucose distribution volume, correlated positively to blood glucose, suggesting sensitivity to insulin. In conclusion, hepatic and splenic metabolic activities are linked, and a possible mechanism could be based on insulin sensitivity. Among other findings, this study confirmed that spleen size is increased in NAFLD [40]. In a further study, NAFLD patients showed a relatively high prevalence of spleen enlargement (splenomegaly,) which was significantly associated with a reduced lysosomal acid lipase (LAL) activity [41]. LAL plays a key role in the regulation of intracellular lipid homeostasis.

To make the matter more complex, the spleen and the liver show different patterns concerning resistivity, evaluated by an ultrasound-based non-invasive technique. Splenic artery resistive index was increased while hepatic artery resistive index was significantly reduced in severe NAFLD [42]. In a study in NAFLD patients with metabolic syndrome, it was confirmed that both the reduction in portal vein flow velocity and vasodilation of intrahepatic arteries were a compensatory way to restore the liver circulation, but the increased spleen volume was likely linked to the organomegaly typical of obese patients [43]. Verifying whether peroxisome proliferator-activated receptor-gamma signaling might play a key role in affecting metabolic pathways through promoting the regulatory cell response of spleens, pioglitazone treatment increased visceral adipose tissue (VAT) weight, reduced serum total cholesterol and hepatic steatosis, but also enhanced ST2^+^ Tregs and Bregs in the VAT and spleen of HFD-fed mice. Specifically, pioglitazone treatment increased IL-10 (antiinflammatory cytokine) in the livers or VAT of HFD-fed mice. The expression of IL-10 in the liver was inversely related to liver weight or serum total cholesterol in pioglitazone-treated HFD-fed mice [44].

In summary, there are many studies reporting spleen to be central in the development of NAFLD, but different mechanisms can be involved. A study aiming at elucidating how the spleen affects the development of NAFLD in a rat model showed that the spleen determines a downregulation of hepatic expression of phosphatase and tensin homolog (PTEN). Splenectomy increased serum lipids, except triglycerides and high-density lipoprotein, in animals fed either a high-fat or normal diet. This protein is important because it results in inhibition of the protein kinase B signalling pathway, which plays an important role in regulating cellular characteristics such as cell growth, survival, and migration [45]. To make things more complex, splenectomy significantly accelerated hepatic steatosis [46].

In the context of well-known chronic low-grade inflammation, a study showed a correlation between spleen size, estimated as SLD, and the duration of psoriasis. In this study, although SLD of control obese patients was larger than that of psoriatic subjects, BMI predicted the volume of the spleen in psoriatic patients. Interestingly, the SLD of psoriatic patients with normal weight was significantly reduced compared to the overweight/obese psoriatic patients. At multiple regression analysis of BMI was the best predictor of spleen volume. Furthermore, the disease duration predicted the spleen size in psoriatic subjects [47]. Irisin reduced body weight and whole-body fat mass in HFD-induced obesity mice, in a dose dependent manner, because of a marked increase in total energy expenditure. This factor decreased blood glucose, insulin, and serum lipid concentrations and eventually reversed hepatic steatosis. Irisin ameliorated both hepatic and peripheral insulin sensitivity. Gene expression profiles of different biomarkers in spleen lend credence to a central role of irisin in inflammation [48].

## 7. Probiotics/Prebiotics

A search for simple theories is a requirement of the research strategy. When theories grow too complex, scientists should apply the so-called Ockham’s Razor, the principle of parsimony.

This should be the case for the very complex microbiota alterations in the pathogenesis of illnesses. Effects of probiotics on body weight, body mass index, fat mass, and fat percentage in subjects with overweight or obesity commonly use high-throughput sequencing platforms [49] to better understand what represents a ‘healthy” gut microbiota. One of the most striking findings is that the resident microbiota encodes >100-fold more genes than the human genome. Research on the human microbiome has proved that even healthy individuals differ remarkably in the microbes that inhabit the gut. Much of this diversity remains unexplained, although early microbial exposure has always been associated [50].

The genes present in the microbiome are responsible for many functions necessary to host survival. Thus, taking into account the vast amount and appropriateness of the metabolic processes carried out by the microbiome, it has been appealed as “our hidden organ” [51]. Recently, it can be stated that the gut microbiota impact on three levels, i.e., host metabolism, nutrient absorption, and immune function. As a consequence, the disruption of this community or alteration of this balanced complex might generate deleterious health consequences. Altered intestinal microbiota, also called dysbiosis, could incentivize hepatic fat storage through several mechanisms, the first being the regulation of gut permeability, but also the modulation dietary choline metabolism, the controlling of bile acid metabolism, the boosting of low-grade inflammation, and the production of endogenous ethanol, all of which might represent fundamental mechanisms [52].

In fact, the authors found that high-alcohol-producing Klebsiella pneumoniae was associated with up to 60% of individuals with NAFLD in a Chinese cohort [53].

Many attempts have been made to try to reverse gut flora modification. Interestingly, administration of probiotics resulted in a significantly larger reduction in body weight expressed as BMI compared to placebo; however, the effect was small. In contrast, the effect of probiotics on fat mass was non-significant [54]. With obesity being the main driver of NAFLD, it is interesting to evaluate the effects of the induced gut flora modification in obese patients. Recently, authors in a placebo-controlled study, verifying the effects of rifaximin treatment on insulin sensitivity, adipose inflammation, and various metabolic parameters, demonstrated that rifamixin treatment for modifying microbiota did not ameliorate metabolic balance in obese patients [55]. In another study, reduction of triglycerides was more significant in the probiotic treatment group than in the placebo group. However, the changes in the percentage of intrahepatic fat fraction and triglyceride levels were not different between placebo and control groups after adjusting for changes in body weight [56].

Indeed, in a placebo-controlled pilot study, authors found that fecal microbiota transplantation capsules (collected from a lean donor) were safe but did not reduce BMI in obese and metabolically unhealthy patients, an concluded it to be an optimistic, but not real, strategy to fight obesity and metabolic syndrome [57]. Accordingly, a systematic review with a meta-analysis failed to confirm the therapeutic efficacy of the fecal microbiota transplantation on obesity and metabolic syndrome [58].

A serious problem is the safety of administering prebiotics and probiotics in humans. Harms reporting in published RCTs assessing probiotics, prebiotics, and synbiotics is often lacking or inadequate, for which reason researchers cannot broadly conclude that these interventions are safe [59]. Gut flora can be modified by various factors, e.g., sex, diet, exercise, as well as drugs. In fact, experts screening more than 1000 marketed drugs against 40 representative gut bacterial strains discovered that 24% of the drugs targeting human diseases of all therapeutic classes inhibited the growth of at least one strain in vitro [60]. Despite evidence of commensal immune system interactions, how these relationships are modified by commensal bacteria colonisation in different compartments of the gastro-intestinal tract is still unclear. Therefore, assessing regulatory mechanisms regarding proper anatomical containment of commensal bacteria is mandatory to save tissue homeostasis and avoid diseases [61].

Recent research has focused mainly on gut bacteria. However, the human gut contains a vast collection of viruses, mostly bacteriophages. The majority persist uncharacterized, and their roles in shaping the gut microbiome and impacting on human health remain scarcely characterized [62].

As collateral findings, a number of large National Heart Lung and Blood Institute-funded trials evaluating drugs or dietary supplements for the treatment or prevention of CV disease found the main complication of obesity-related NAFLD has increased over time, but those trials reporting significant results fell after the year 2000 [63]. Accordingly, authors found that published trials on the use of prebiotics and probiotics in curing NAFLD lack consistency [64]. As a final consideration, there is a link between gut flora and the spleen. The presence of a less diverse microbiota with a higher selection of pathogenic species ends up in the quick refilling of the marginal zone and the IgM plasma cell compartment of the spleen, as well as of IgA plasma cells in the gut. Spleen development is modulated by neonatal gut microbiota [65]. In this regard, the regeneration of organs which hide functional white pulp tissue could provide an opportunity for efficacious immunotherapy against various diseases [66].

## 8. Animal Models

Indeed, it should be emphasised that the translation of results obtained in animal models for NAFLD to the equivalent human disease has consistently failed, although those models have contributed to comprehending many mechanisms, and hence show their relevance in this field. Principal animal models of NAFLD/NASH are shown in Table 2.

### 8.1. MCD Diet

The methionine and choline deficient (MCD) diet has a consistent sucrose content (nearly 40%) and limited fat content (nearly 10%). The mechanisms at the basis of this model deficiency consist of impaired β-oxidation and altered secretion of very low-density lipoprotein particles [67], ending up in oxidative stress, apoptotic cell death, hepatic fat accumulation, and the release of cytokines and adipokines—all of these events similar to human NASH.

The weakness of the MCD is the partial lack of metabolic features that are characteristic of human NAFLD, i.e., IR, dyslipidemia, and eventual obesity, casting doubts on its validity to comprehend the complex aspects of NAFLD.

### 8.2. High-Fat Diet

There is no uniform content in the HFD due the fact that multiple regimens (with different fat contents) have been suggested. The HFD brings a phenotype quite similar to the human disease, characterized by obesity, dyslipidemia, and IR [68], but leads to only minimal fibrosis even though the animals are fed for long time.

### 8.3. Genetic Modifications

*Leprdb/Leprdb* (db/db) mice hold a natural mutation in the leptin receptor gene, ending up in no function. Thus, these mice show a similar phenotype to that of the ob/ob mice, even though they have normal to increased leptin levels [69]. A similar mutation is present in rats and is known as *Leprfa/Leprfa* (fa/fa, also called Zucker rats). These rats show overlapping phenotypes to both ob/ob and db/db mice, as they spontaneously develop severe obesity, IR, and hepatic steatosis.

Furthermore, these animals are characterized by hyperleptinemia, and are hyperphagic and generally inactive. Both db/db mice and fa/fa rats do not naturally progress to NASH, and require a further stimulus, as do ob/ob mice [70]. Sterol regulatory element-binding protein-1c transgenic mice display severe IR and NASH with perivenular and pericellular fibrosis, but show reduced adipose tissue mass [67]. A successful study using an interesting animal model was carried out by Lee et al., who found evidence that in transgenic mice, fed a HFD, hepatic triglyceride levels do not arise from augmented hepatic de novo lipogenesis, decreased hepatic free fatty acid oxidation, or decreased very-low-density lipoprotein secretion, establishing that retinol binding protein 4 (RBP4) is expressed in adipocytes and induces hepatic steatosis as a consequence of the principal effects occurring in adipose tissue [71].

To test the validity of this model, dealing with RBP4 in NAFLD patients, serum RBP4 was significantly lower compared to controls and did not correlate to IR. In contrast, RBP4 liver tissue expression was increased and was in line with NAFLD histology [72].

## 9. Antioxidant Therapy, Vitamin D Supplementation, and Blood Lipid-Lowering Therapy

Adopting antioxidant therapy as a treatment for oxidative stress-related diseases such as CV disease, T2DM, or obesity-related diseases, i.e., NAFLD is still arguable. In fact, in a study in which C57BL/6J mice were fed a high-fat diet for 14 weeks, with (OE group) or without (O group) vitamin E supplementation, O mice presented with mild obesity, but it was not sufficient to develop metabolic alterations or oxidative stress. These animals showed a natural enlargement of retroperitoneal WAT (rpWAT). In this rpWAT, they presented a reduced generation of ROS. Meanwhile, the liver did not show signs of lipotoxicity. It is worth noting that despite obtaining a similar body weight, OE mice were insulin resistant. The aforementioned research in vivo highlights the role of ROS as second messengers in adipogenesis, lipid metabolism, and insulin signaling. Decreasing ROS generation below physiological levels when the oxidative process has not yet been completed could be the origin of the questionable results obtained by antioxidant therapy [73]. Previous observational studies have established a correlation between vitamin D (VitD) deficiency and NAFLD, even though this research could not show any causality between these two cases. Researchers systematically and critically reviewed the available clinical trials to clarify these associations, selecting a total of six studies. Only in two studies was the grade of hepatic steatosis clearly reduced after vitamin D supplementation. IR parameters changed significantly in only one. Of the three included studies that measured biomarkers of oxidative stress and inflammation, one showed a significant reduction of these biomarkers after VitD supplementation [74].

As previously emphasized, the effect of VitD supplementation on metabolic syndrome remains controversial. In a prospective, randomized, open-label, blinded end-point designed study, fifty patients with metabolic syndrome were enrolled and randomized either to dietary instructions (control group) or dietary instructions plus VitD 2000 IU/day for three months. In both groups, a similar small weight reduction was achieved. In the VitD group, serum 25(OH) VitD levels were significantly augmented by 91%, while in the control group no clear change was detected. In both groups, triglycerides, HDL cholesterol, LDL cholesterol, fasting glucose, haemoglobin A_1c_, HOMA index, and diastolic blood pressure did not differ. Systolic blood pressure was not reduced by a mere 3.7% in the VitD group. In conclusion, VitD supplementation did not impact on CV disease risk factors in patients with metabolic syndrome [75].

Still, genetically lowered 25(OH) D levels were not related to an increased risk of coronary artery disease in a large, well-powered study, suggesting that previous associations between circulating 25(OH) D levels and coronary artery disease (CAD) are due to confounding factors or to reverse causation [76].

In contrast with previous observations, the aim of a recent study was to systematically evaluate the association of VitD levels, as measured by serum 25(OH) D, with NAFLD and NASH. In total, twenty-nine articles met the eligibility criteria (24 studies about NAFLD and four studies about NASH) and were included in the meta-analysis, comprising of cross-sectional and case-control studies. In comparison to controls, the NAFLD patients had significantly lower levels of 25(OH) D and were 1.26 times more likely to be VitD deficient. Similarly, the NASH patients had significantly decreased levels of 25(OH) D. Although the cross-sectional studies did not allow the establishment of a net causality, this meta-analysis showed lower serum 25(OH) D levels in NAFLD and NASH patients than in subjects without NAFLD or NASH, hypothesizing that hypovitaminosis D likely has a role in the pathogenesis of NAFLD and NASH [77]. This hypovitaminosis D in NAFLD patients could be explained by the observation that NAFLD is mostly linked to obesity. Indeed, a correlation between VitD and obesity could also be due to the fact that obese people are less likely to get exposure to the sun. In fact, VitD is likely a surrogate marker for lack of chronic sun exposure, which is a risk factor for non-melanoma skin cancers [78].

Recently, it was reported that statins decreased the hepatic expression of perilipin 5 (Plin5), a lipid droplet (LD)-associated protein, which regulates lipid accumulation and lipolysis in liver. On this basis, authors showed that atorvastatin moderately decreased the expression of Plin5 in the livers of of mice fed HFD without changing the protein level of Plin5 in the hepatic LD fraction. Interestingly, atorvastatin stimulated the PKA-mediated phosphorylation of Plin5 and reduced the triglyceride (TG) storage in hepatocytes with overexpression of wide type (Plin5-WT). Glucagon, a PKA activator, stimulated the phosphorylation of Plin5-WT. The results indicated that atorvastatin induced lipolysis and decreased TG storage in the liver by increasing PKA-mediated phosphorylation of Plin5 [79].

These data indicate that statins could be a valuable therapeutical option when dealing with NAFLD and NASH patients. Obviously, this should be cautiously established because the evidence is limited to post hoc analyses of clinical studies and is based on reduced numbers of patients with biopsy-proven diagnosis of NAFLD or NASH [80].

Surprisingly, a recent meta-analysis showed that statin use was significantly related to a decreasd risk of virus-related cirrhosis and decompensation [81].

## 10. Macrophage and Chronic Low-Grade Inflammation

Obesity-induced metabolic disease involves an interplay among several organs via circulating factors, allowing crosstalk between the liver and VAT. In obesity, inflammatory adipose tissue macrophages indwell VAT. In obese humans, adipose tissue is strongly related to inflammation and IR. In fact, blocking systemic inflammation due to adipose tissue macrophage infiltration in obese mice ameliorates insulin sensitivity. Adipose tissue macrophages are believed to arise from bone marrow-derived monocytes, which infiltrate the tissue from the blood stream. Authors showed that macrophages within VAT, as well as in the liver and the spleen, displayed increased proliferation in obesity. Treatment with monocyte chemotactic protein 1 (MCP-1) induced macrophage cell division in adipose tissue explants, while MCP-1 deficiency in vivo reduced the proliferation of adipose tissue macrophages [82].

## 11. The Main Role of Transforming Growth Factor-Beta

Collagen forms fibrotic tissue in the liver. Fibronectin is central to collagen matrix assembly in vitro. It also modulates the amount of growth factors and their release from the matrix. Accordingly, authors examined the effects of the absence of fibronectin on the development of fibrosis in mice. They observed that the boosted fibrosis in fibronectin-deficient mice was associated with enhanced stellate cell activation and proliferation, elevated concentrations of active transforming growth factor-beta (TGF-β), and increased TGF-β-mediated signalling [83]. NAFLD progression towards NASH is determined by chronic low-level type 1 inflammation originating in the adipose tissue during obesity, however the specific immunological mechanisms are unclear. Recently, authors investigating fibrosis in mice found that IFN-gamma-deficient mice progressed rapidly to NASH with evidence of fibrosis dependent on TGF-β and IL-13 signalling. Simultaneous inhibition of TGF-β and IL-13 signalling attenuated the fibrotic machinery more completely than TGF-β alone in NAFLD-associated fibrosis [84].

Although TGF-β is one of the most potent stimuli of extracellular matrix synthesis, suppressing its expression remains a major challenge of antifibrotic therapy, since systemic blocking of TGF-β can provoke inflammation and could increase the risk of neoplasia. Different research casts doubts on this sequela. In fact, neutralization of TGF-β in animal models inhibits liver fibrosis and reduces the risk of developing cholangiocarcinoma [85]. As a TGF-β target gene, connective tissue growth factor (CTGF) is considered a central mediator, which is specific for promoting fibrogenensis. It has been reported that inhibition of CTGF expression by siRNA prevents CCl4-induced liver fibrosis and can induce the regression of liver fibrosis [86].

The finding that Kupffer cells are stimulated to produce TGF-β, which activates hepatic stellate cells, fibrocytes, and mesenchymal stem cells into myofibroblasts, highlights another aspect, about which researchers are still debating, i.e., could fibrosis alone make simple fatty liver progress towards a more severe form (i.e., advanced fibrosis/liver cirrhosis) without the mediation of inflammation?

In keeping with this hypothesis, authors showed that high concentrations of TGF-β1 were found in patients suffering from both fatty liver and NASH, suggesting that these forms share more common aspects regarding their progression than previously thought [87].

## 12. New and Alternative Mechanisms

Lead exposure impacts on lipid peroxidation, overproduction of ROS, and reduction in the activity of antioxidant enzymes, such as superoxide dismutase, catalase, and glutathione peroxidase in hepatocytes [88].

Metabolic syndrome increases the risk for CV disease and T2DM. The worldwide increase in the prevalence of metabolic syndrome is typically ascribed to lifestyle factors such as sedentary behavior and caloric intake. Alternative mechanisms have been proposed.

Indeed, environmental exposures such as heavy metals have been involved even though results are conflicting and possible mechanisms remain unclear [89]. Authors found that Pb2+ exposure could be a lethal disruptor of lipid metabolism in hepatocytes, and highlighted sorcin (a cytosolic adaptor partner of carbohydrate responsive element binding protein (ChREBP)-dependent hepatic lipogenesis) as a novel therapeutic target against Pb2+-induced hepatic dyslipidemia [90]. Hepatic de-novo lipogenesis (DNL) is a metabolic process proposed in the pathogenesis of T2DM, with multiple enzymes involved in this process [91].

To test that DNL is a central abnormality in NAFLD, authors showed that lipogenic enzymes and the glucose transporter-4 are markedly decreased in WAT of insulin-resistant obese individuals compared with non-obese controls. By contrast, lipogenic enzymes are substantially upregulated in the livers of obese subjects; among them the ChREBP plays a key role [92]. The isotope analysis was used to compare DNL and fatty acid flux in obese subjects with and without NAFLD. The proportion of hepatic TG deriving from the evening meal was quite similar at the completion of research. The proportion of TG synthesised from plasma Free Fatty Acid (FFA) in low liver-fat group was significantly higher than in high liver-fat group. In contrast, TG synthesized via DNL were significantly higher in the high liver-fat group compared to the low liver-fat group [93]. In another study, authors showed that inhibition of DNL in obese subjects, unless coupled with a correction of the chronic positive energy balance, could consequently induce lipotoxicity and metabolic stress. In contrast, strategies aimed at specifically activating DNL in adipose tissue could contribute to supporting metabolic homeostasis in obese subjects [94].

Bilirubin may protect against insulin IR, the main driver of NAFLD, by ameliorating visceral obesity and adipose tissue inflammation [95].

Direct bilirubin reduced NAFLD risk independent of possible confounders among a middle-aged and elderly Chinese population, probably based on the endogenous antioxidation of bilirubin [96]. There is still controversy about what type of serum bilirubin is implicated in NAFLD pathogenesis. In fact, some authors reported that the level of serum conjugated bilirubin was related to the occurrence of NAFLD and others reported that the level of serum unconjugated bilirubin was related to the severity of NAFLD [97,98,99].

Aquaporin (AQP) 7 and AQP9 are subcategorized as aquaglyceroporins that transport glycerin in addition to water. These AQPs are believed to play a role in the homeostasis of energy metabolism. Investigators studied the effect of AQP7, AQP9, and lipid metabolism-related gene expression in diet-induced obese (DIO) mice, characterized by excess lipid stored in the liver. Hepatic AQP9 gene expression was significantly increased in DIO compared to controls. The mRNA expression levels of fatty acid and TG synthesis-related genes and fatty acid β-oxidation-related genes in the liver were increased in this mouse model, suggesting that TG synthesis in this organ results in glycerol release from adipocytes. Furthermore, adipose AQP7 and AQP9 gene expressions were increased in DIO mice [100].

Processes promoting adipose tissue inflammation in obesity have been studied for long time. Recently, authors demonstrated that obesity in mice stimulates hepatocytes to synthesize and secrete dipeptidyl peptidase 4 (DPP4), which acts with plasma factor Xa to inflame adipose tissue macrophages. Silencing expression of DPP4 in hepatocytes restrains inflammation of VAT and IR; however, a similar effect is not seen with the orally administered DPP4 inhibitor sitagliptin. Inflammation and IR are also overcome by silencing expression of caveolin-1 or PAR2 in adipose tissue macrophages, hypothesizing that these proteins act as mediators of DPP4 and factor Xa [101].

As repeatedly stressed, T2DM is one of most common metabolic disorders linked to NAFLD. Researchers have shown that the Let-7 family of microRNAs regulates glucose metabolism in multiple organs. In fact, global and pancreas-specific over-expression of Let-7 in mice resulted in impaired glucose tolerance and reduced glucose-induced pancreatic insulin secretion. Global knockdown of the Let-7 family with an anti-miR was apt to anticipate and treat impaired glucose tolerance in mice with diet-induced obesity, at least in part by improving insulin sensitivity in liver and muscle [102].

A recent meta-analysis provides strong epidemiological evidence for the relationship between hypothyroidism and NAFLD. Both patients with subclinical and overt hypothyroidism are at major risk for NAFLD than euthyroid subjects [103].

Because adipose tissue gives place to resident macrophages that have been implicated in the generation of IR, i.e., in expanding fat mass, the authors verified whether adipocytes release factors that impact on angiotensin-converting enzyme (ACE) expression and function in monocytes could have a function. Incubation of human monocyte-derived macrophages with conditioned medium from freshly isolated human adipocytes resulted in a four-fold increase in ACE expression. Interestingly, human macrophage ACE expression was also up-regulated by IL-4 and IL-13, which promote the “alternative” activation of macrophages and decrease IFN-gamma [104].

Genetic- and diet-induced obesity and IR both play a key role in NAFLD and are associated with an increase in mechanistic target of rapamycin complex (mTORC) 1 activity in adipose tissue. Although rapamycin did not modify body weight, it worsened the IR and adipose tissue inflammation induced by HFD feeding, as demonstrated by the augmented adipose tissue percentage of M1 macrophages, naive and activated cytotoxic T lymphocytes, and mRNA levels of pro-inflammatory molecules, such as the always involved IL-6, TNF-α, and MCP-1 [105].

In light of the high capacity for mitochondrial fatty acid β-oxidation, authors tried to understand the role of this oxidative process in response to a chronic HFD, generating mice with a liver-specific deficiency of mitochondrial long chain fatty acid β-oxidation (Cpt2L−/− mice). Paradoxically, Cpt2L−/− mice were resistant to HFD-induced obesity and IR without liver injury, although they showed serum dyslipidemia, hepatic oxidative stress, and systemic carnitine deficiency [106].

NAFLD severity increases with ageing. Ageing hallmarks that negatively impact health include weight gain and reduced physical activity, which can increase IR. To try to clarify the underlying mechanism, authors found that ageing increases DNA breaks and activates DNA-dependent protein kinase (DNA-PK), which phosphorylates threonines 5 and 7 of HSP90α by decreasing its chaperone function for clients such as AMP-activated protein kinase (AMPK). AMPK is critical for mitochondrial biogenesis and energy metabolism [107]. Caloric restriction has been shown to increase longevity in various organisms. This effect has been associated with a decreased fat mass and alterations in the insulin/insulin-like growth factor 1 (IGF-1) axis. Authors studying mice with a fat-specific insulin receptor knockout found that these animals have reduced fat mass and are protected against age-related obesity, although their food intake was normal [108].

It has been claimed that glucose-rich diets shorten longevity [109], likely due to the high glycemic index of some foods [110]. Still, mendelian randomization analyses have clearly shown a causal relationship of glucose-stimulated insulin secretion with body weight, according to the carbohydrate–insulin model of obesity [111].

Serum concentrations of keratins 8/keratins 18 (K8/K18) were found increased in the severe form of NAFLD compared to those of the simple fatty liver [112]. K8/K18 are both constituents of hepatocytes, functioning as epithelial cell intermediate filament proteins, meaning that a K8 loss in K8-null mice leads to degradation of K18. Only recently did authors investigate the role of K8/K18 in the regulation of insulin receptor signalling and trafficking in hepatocytes, finding that the insulin receptor substrate 1 (IRS1)/PI3K/Akt signalling cascade shows prolonged activation in K8-null compared with wild-type hepatocytes. Assessment of the Akt/mammalian target of rapamycin complex 1-mediated feedback loop to IRS1/PI3K further supports a preferential K8/K18 IF role at trafficking in hepatocyte [113].

Recent results highlighted that miR-876-3p regulates glucose homeostasis and its dysregulation leads to IR. Investigators found that miR-876-3p levels are a critical determinant to adiponectin expression by virtue of its target within adiponectin 3′UTR. These findings suggest that modulating miR-876-3p expression could provide novel opportunities for therapeutic intervention of obesity-associated metabolic syndrome [114].

Intramyocellular lipid (IMCL), also called intramuscular triglycerides, accumulation has been linked to IR and has been found related to the severity of NAFLD [115]. The association of IR with accumulation of saturated IMCL is consistent with (j) a potential pathogenic role for saturated fat, and (jj) the reported benefits of exercise and diet in insulin resistant states [116].

Dealing with the main role of monounsaturated fatty acid (MUFA), authors generating mouse models with restricted exogenous MUFA supply and reduced endogenous MUFA synthesis (in which stearoyl-coenzyme A desaturase-1 global knockout or liver-specific knockout mice were fed a lipogenic high-sucrose very low-fat or high-carbohydrate diet), revealed that hepatic availability of oleate (one of the MUFAs) is important for maintenance of liver health [117]. SCD1 is a delta-9 fatty acid desaturase that converts saturated fatty acids into MUFA and this activity is elevated by dietary carbohydrate. Dietary fat restriction, together with high carbohydrate intake, as previously mentioned, increases endogenous fatty acid synthesis. Dietary and endogenously synthesized fatty acids both contribute to the whole-body fatty acid pool. Thus, obesity can therefore result from excessive fat or carbohydrate consumption. Mice lacking *Scd1* are protected from obesity and IR and are characterised by decreased fatty acid synthesis and increased fatty acid oxidation [118]. Contextually, other researchers demonstrated that hepatic de novo synthesized oleate, but not palmitoleate, stimulate hepatic lipid accumulation and adiposity, counteracting the protective effect of the global *Scd1* knockout under lipogenic conditions [119].

## 13. Unanswered Questions

It is conflicting to propose a new interpretation against a well-accepted one, i.e., that NAFLD/NASH is a mere pathological and dangerous consequence of calorie-rich foods and sedentary lifestyle. This paper is based on a completely different mechanistic approach that focuses on a fascinating hypothesis: NAFLD is an adaptive physiological process in response to unexpected and new conditions which the human organism undergoes, at least in the early phases, like the onset of type 2 diabetes mellitus in response to famine or the presence of Mediterranean anaemia in endemic area of malaria.

Another interesting question is: Could hepatocytes be considered last adipocytes? In the sense that when the expansion of the adipose tissue that is genetically determined is at a halt, the liver acts as an ultimate and more powerful defence of the organism to a hostile environment for which it is not prepared.

Still, is the paradigm of “more fat means more metabolic disease” correct? Some subjects, referred to as metabolically healthy obese, have long-lasting obesity and obesity graded as severe, but can be considered healthy despite their high degree of obesity [120].

Moreover, are non-obese NAFLD patients really non-obese [121]? Finally, coming back to a previously mentioned dilemma, does simple fatty liver remain forever as a benign form of NAFLD?

## 14. Future Directions

Scientists and care providing specialists should completely change their approach to NAFLD, focusing on the causes that determine the initial and lighter forms of NAFLD, which could be considered as a response not a disease, instead of being obsessed with the diagnosis and therapy of the more severe form of NAFLD, i.e., NASH, to break the vicious circle between the causes and consequences of NAFLD/NASH.

Furthermore, it is necessary to develop evidence that changes in climate, agro-alimentary logistics, and political direction play a central role in favouring the presence of NAFLD, making the issue more and more complex.

## Figures and Tables

**Table 1:** The controversial role of the spleen: Inducer or protective organ of non-alcoholic fatty liver disease (NAFLD)?

Studies in Favour of Spleen as an Inducer			Studies in Favour of a Protective Effect of the Spleen		
Authors	References	Type of Study (in)	Authors	References	Type of Study (in)
Tarantino G, et al.	[29]	Humans	Inoue M, et al.	[37]	Rats
Suzuki K, et al.	[32]	Humans	Tana C, et al.	[42]	Humans
Zardi EM, et al.	[33]	Humans	Soresi M, et al.	[43]	Humans
Murotomi K, et al.	[34]	Mice			
Nitta CF, et al.	[36]	Isolated cells			
Tarantino G, et al.	[39]	Humans			
Keramida G, et al.	[40]	Humans			
Polimeni L, et al.	[41]	Humans			

**Table 2:** Animal Models.

GENETIC MODELS
SREBP-1c transgenic mice
Ob/ob mice
Db/db mice
KK-Ay mice
PTEN 10 null mice
Peroxisome proliferator-activated receptor-α knockout mice
Acyl-coenzyme A oxidase null mice
Methionine adenosyltransferase-1A null mice
DIETARY MODELS OF NASH
Methionine and choline deficiency;
HFD
Cholesterol and cholate
Fructose
COMBINED MODEL OF GENETIC MODIFICATION AND NUTRITIONAL/DIETARY CHALLENGE
MCD diet to ob/ob and db/db mice
PPAR-α null mice fed an MCD diet
HF in Alms 1 Mutant b mice
Male and female hyperlipidemic (low-density lipoprotein receptor-deficient [ldlr(-/-)] and Apolipoprotein E2 knock-in [APOE2ki]) mouse models

## Abbreviations

NAFLD	nonalcoholic fatty liver disease
NASH	nonalcoholic steatohepatitis
T2DM	type 2 diabetes mellitus
ROS	reactive oxygen species
IR	insulin resistance
CV	cardiovascular
LAGB	laparoscopic gastric banding
PNPLA	patatin-like phospholipase domain-containing protein 3
HFD	high fat diet
WAT	white adipose tissue
EDC	endocrine disrupting compound
HIF	hypoxia inducible factor
TSK	Tsukushi
SL	spleen longitudinal diameter
LPS	lipopolisaccaride
SPX	splenectomy
MRglu	metabolic rate of glucose
CT	computerised tomography
LAL	lysosomal acid lipase
VAT	visceral adipose tissue
MCD	methionine and choline deficient
VitD	vitamin D
TG	triglycerides
LD	lipid droplet
MCP-1	monocyte chemotactic protein 1
ChREBP	carbohydrate responsive element binding protein
DNL	hepatic de-novo lipogenesis
AQP	aquaporin
DIO	diet-induced obese
DPp4	dipeptidyl peptidase 4
ACE	angiotensin-converting enzyme
mTORC	target of rapamycin complex
DNA-PK	DNA-dependent protein kinase
AMPK	AMP-activated protein kinase
IGF-1	insulin-like growth factor 1
K8/K18	keratins 8/keratins 18
IRS1	insulin receptor substrate1
IMCL	intramyocellular lipid
MUFA	monounsaturated fatty acid
TGF-β	transforming growth factor-beta
